# Prevalence of Metabolic Syndrome According to Absolute and Relative Values of Muscle Strength in Middle-Aged and Elderly Women

**DOI:** 10.3390/ijerph18179073

**Published:** 2021-08-27

**Authors:** Wangyang Zhang, Zijian Zhao, Xuebin Sun, Xiaoxia Tian

**Affiliations:** 1 School of Physical Education in Main Campus, Postdoctoral Mobile Station of Public Administration, Zhengzhou University, Zhengzhou 450001, China; zwyzzu@zzu.edu.cn; 2 School of Physical Education in Main Campus, Zhengzhou University, Zhengzhou 450001, China; zjzhao@zzu.edu.cn; 3 Department of Education, Woosuk University, Wanjun 55338, Korea

**Keywords:** hand grip strength, leg strength, prevalence, metabolic syndrome

## Abstract

Metabolic syndrome (MetS) increases with age, obesity, low physical activity, and decreased muscle strength. Although many studies have reported on grip strength and MetS, few studies have been conducted on leg strength. The purpose of this study was to analyze the prevalence of MetS according to absolute and relative leg strength values in middle-aged and older women. The participants were 1053 women who visited the healthcare center: middle-aged (*n* = 453) and older (*n* = 601). MetS was diagnosed using the criteria established by the third report of the National Cholesterol Education Program Adult Treatment Panel III and the World Health Organization’s Asia Pacific guidelines for waist circumference. For leg strength, knee flexion and extension were performed using isokinetic equipment. Grip strength was measured using a grip dynamometer and classified into quartiles. Analysis of prevalence using logistic regression showed that MetS was present in 21.2% of middle-aged and 39.4% of older women. The lowest relative leg extension increased 2.5 times in the middle-aged and 1.5 times in older women (*p* < 0.05). However, leg flexion did not have a significant prevalence in either age group. The prevalence of MetS in middle-aged and older women with the lowest relative grip strength increased 1.5 and 1.2 times, respectively. Conversely, the lower the absolute leg extension strength, the lower the MetS prevalence was at 0.520 in middle-aged and 0.566 in older women (*p* < 0.05). In conclusion, the prevalence of MetS increased in women with low relative grip and leg strengths. Specifically, the lower the relative leg extension muscle strength, the higher the prevalence of MetS. In addition, the prevalence of MetS increased in the high-frequency alcohol consumption and non-physical activity group.

## 1. Introduction

Metabolic syndrome (MetS) is diagnosed when several cardiovascular risk factors are present, which increase the risk of myocardial infarction and stroke [1]. There are various institutions that diagnose MetS, and many share similar diagnostic criteria such as blood pressure, glucose, and obesity [2]. One is the third report of the National Cholesterol Education Program Adult Treatment Panel (NCEP-ATP III), and individuals having three or more of the five factors are diagnosed with MetS: high systolic blood pressure (SBP) or diastolic blood pressure (DBP), high triglyceride (TG) levels, reduced high-density lipoprotein cholesterol (HDLC), high fasting glucose levels, and large waist circumference [3]. 

Patients with MetS have a 1.3 times increased risk of myocardial infarction, irrespective of body mass index (BMI) [4], and a ~2-fold increased risk of stroke [5], compared to those without MetS. Aguilar et al. [6] reported that the incidence of MetS is increasing, and the trend is similar in men and women. Specifically, the incidence of MetS in the United States has increased from 30.9% to 32.8% in men and from 34.7% to 36.6% in women over the past decade. In Korea, however, the prevalence of MetS in men increased from 27.9% in 2008 to 30.8% in 2013, but remained relatively constant at ~26% in women over the same period [7]. Likewise, a Chinese study reported that the prevalence of MetS in men and women was similar at 50.99% and 49.01%, respectively [8]. 

MetS risk factors include non-modifiable factors such as age and heredity, and modifiable factors such as obesity, nutrition, and low physical activity [1]. In particular, low physical activity causes a decrease in muscle strength, which is known to increase MetS [9,10,11]. The prevalence of MetS increased 2.7-fold in women belonging to the quartile with lowest grip strength compared to the group in the highest quartile [9], and the prevalence of MetS increased 4.7-fold in men with low relative grip strength [10]. In a study of leg strength, MetS increased by 1.23 times in men with low muscle strength [11], and in another study, MetS increased up to 3.3 times in men with low leg extension strength [10]. Most of these studies calculated relative strength by dividing the measured strength by body weight. Muscle strength tends to be proportional to body weight; therefore, overweight individuals may exhibit a higher incidence of MetS and higher muscle strength [9]. Therefore, previous studies suggested that the relative value is more appropriate for MetS analysis [9,12].

Although there are many studies on grip strength, few studies have examined leg strength. This is because the measurement of grip strength is more convenient and less expensive than leg strength measurement [13]. Isokinetic equipment is accurate enough to be regarded as the gold standard tool for muscle strength evaluation and has high reliability and safety. However, the equipment is expensive and less portable. Additionally, the measurement procedure is more complicated than that for measuring grip strength. Therefore, analysis using isokinetic equipment is rare in MetS studies [14,15]. 

This study was conducted to include the following characteristics. Although this was a cross-sectional study, the prevalence of MetS based on absolute and relative strengths was analyzed. Leg muscle strength was measured using isokinetic equipment, and middle-aged and older women were the participants. We hypothesized that in women, the lower the absolute strength, the lower the prevalence of MetS, and the lower the relative strength, the higher the MetS.

## 2. Methods

### 2.1. Participants

**Figure 1:**
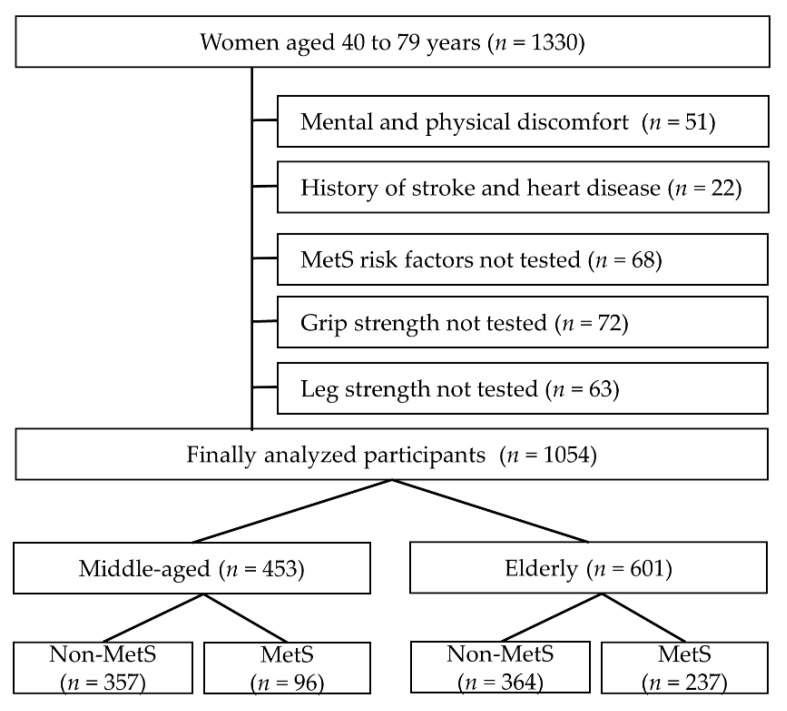
Participants’ inclusion and exclusion diagram.

Participants had visited a healthcare center at a hospital for preventive purposes during 2017–2018, and had no severe disease. The sample size was calculated using G*power software (G*power 3.1, University of Düsseldorf, Düsseldorf, Germany): z test and logistic regression, Pr(Y = 1|X = 1) H0 = 0.1, α error = 0.05, power (1-β err prob) = 0.90, and R2 other X = 0. Therefore, the number of women in the 40- to 79-year age group initially involved in this study was 1330. We excluded patients with a history of heart disease or stroke (*n* = 22), whose grip strength was not measured (*n* = 72), whose isokinetic leg strength was not measured (*n* = 63), and MetS patients who did not complete the test (*n* = 68). Patients who were unable to perform strength or other tests due to mental and physical health issues (*n* = 51) were also excluded. Finally, 1054 participants were included: middle-aged (40–59 years, *n* = 453) and older (60–79 years, *n* = 601) (Figure 1).

Study participants were required to fast for eight hours before arrival at the laboratory between 8:00 am and 12:00 pm. All participants were provided with similar clothing and shoes as required for the examination. Participants completed a health-related questionnaire. A medical specialist, nurse, or physical fitness expert took participants’ body measurements and conducted blood pressure, blood, and strength tests. 

This study complied with the guidelines of the Helsinki Declaration, and was conducted ethically and monitored by a supervisory authority. The study included only participants who provided written consent after the purpose, method, and procedure of the study were explained. The participants submitted written consent forms. This study was approved by an affiliated institution (ZZUIRB 20210310).

### 2.2. Diagnosis of Metabolic Syndrome (MetS)

MetS in women was diagnosed on the basis of the presence of three or more of the five criteria of the National Cholesterol Education Program (NCEP) Adult Treatment Panel-III (NCEP-ATP III) [3]. These included SBP ≥ 130 mmHg and/or DBP ≥ 85 mmHg, TG level ≥ 150 mg/dL, HDLC < 50 mg/dL, and glucose level ≥ 100 mg/dL. The World Health Organization Asia Pacific standard was applied for waist circumference ≥ 80 cm for women [16]. Patients who had already been prescribed medication for hypertension, hyperlipidemia, or diabetes were included as participants with risk factors.

### 2.3. Strength Tests

The patient performed stretching and light exercises to warm-up before the tests. The test was conducted based on the prior literature [17,18,19]. The examiner asked about past or present injury history prior to the examination. Therefore, the healthy side or the preferred side was examined first, and then the opposite side was examined. The grip strength test was performed first, followed by the isokinetic leg test. Absolute strength values were recorded as “kg” for grip strength and “Nm” for leg strength. The relative value (Nm/body weight or kg/body weight) was calculated as the absolute value divided by the body weight.

#### 2.3.1. Grip Strength 

Tests were conducted with reference to the existing literature using a grip dynamometer (TKK5401, Takei, Japan) [17]. Participants looked straight ahead and stood with feet shoulder-width apart, with the waist and chest straight. The arms were slightly spread out so that the hands did not touch the thighs, and the elbows were extended. The sensor gauge was adjusted so that the middle phalanges could hold the handle in an upright position. When the examiner provided the start signal, the individual gripped as firmly as possible while maintaining the specified posture. The grip strength was measured twice for both hands and the maximum value for each hand was recorded. The average of the maximum values for both hands was used for the analysis.

#### 2.3.2. Leg Strength: Extension and Flexion 

Leg extension and flexion were measured to determine leg strength using an isokinetic device (CSMi Humac Norm, Stoughton, MA, USA), according to the manufacturer’s manual and published literature [18,19]. Test guidelines recommend that at least three repetitions be performed for accurate examination on the actual test. The healthy leg or the preferred leg should be tested first; practice should be performed with three to five repetitions of sub-maximum and one repetition of maximum practice. The tester should provide consistent explanations and verbal encouragement to every participant [18,19]. 

The condition of the patient, injury to the knee and lower extremities, and the state at which the maximum strength could be exerted were evaluated before the examination. To enable the subject to gain familiarity with the machine, several movements were performed for practice at low, medium, and high speeds. When the participants became accustomed to the machine, the actual test was performed four times at 60°/s as a concentric contraction. 

The participant sat on the chair of the test equipment with a fixed pad on the trunk. The knee was bent at 90° with the axes of the knees aligned with the femoral lateral epicondyle, and the ankle pads were secured to the distal tibia. The upper body was straightened and the subject leaned against the back of the chair. The pelvis and torso were fixed using pads, straps, and belts, respectively. The handle was held to prevent the upper body from shaking during the test. 

The examination was initiated at an angle of 90° of flexion and completed at 0° of extension. The preferred leg was tested first, and then the muscle strength of the non-preferred leg was subsequently tested. The test was conducted by a skilled individual to ensure accurate examination and was performed to exert maximum extension and flexion strength. 

When abnormal graphs suggested a lack of understanding or machine maladaptation, a retest was performed after sufficient recovery. Previous literature indicate that a rest period of 30–60 s is sufficient for retest [20,21]. Considering that the participants included older women, an extended recovery time of 3 min was provided in this study.

### 2.4. Health Behavior and Medical History Questionnaire

The health behavior questionnaire surveyed alcohol consumption, smoking status, and physical activity. The type, amount, and consumption frequency of alcohol were analyzed in this study. The consumption frequency was classified as none, once per day/month, once per day/week, and >2 days/week. For smoking status, present, quit, and never were investigated first. Although the period and amount of smoking were investigated in those who previously smoked, the current smoking status was used for the analysis. Physical activity was assessed based on duration, frequency, and intensity, and the prevalence was analyzed using weekly frequency. It was classified as 5–7 days/week, 3–4 days/week, 1–2 days/week, and none. Previously diagnosed diseases and current medication status were evaluated. 

Medical history was recorded on the basis of current medication status. The patients selected “yes” or “no” using a questionnaire. The study investigated whether participants were being managed on antihypertensive drugs, oral hypoglycemic agents or insulin for diabetes, and medications for improving dyslipidemia.

### 2.5. Data Analysis

SPSS 25.0 (IBM SPSS Inc., Armonk, NY, USA) was used for data analysis. Continuous variables of general characteristics were expressed as means ± standard deviations, and MetS and healthy groups were compared. Normal distribution was not shown according to the Kolmogorov–Smirnov test; therefore, the non-parametric Mann–Whitney U-test was used for between-group comparisons. The relationship between medication status, health behavior, and MetS was analyzed using the chi-square test. Grip and leg strength were first classified into quartiles: Q1, Q2, Q3, and Q4. The prevalence was expressed as odds ratio (OR) by logistic regression analysis. The reference group (Q1) exhibited the highest strength, with no alcohol consumption, no smoking, and high physical activity. For the adjustment variables, Model 1 included only age. Model 2 included age, alcohol consumption, smoking, physical activity in OR of strength, and Model 2 included age and strength in OR of alcohol consumption, smoking, and physical activity. The significance level was set at *p* < 0.05, and the confidence interval (CI) was 95% with lower and upper values.

## 3. Results

Table 1 compares the general characteristics of the MetS and non-MetS groups. There were significant differences in weight, BMI, and risk factors for MetS in middle-aged and older women (*p* < 0.001 for all). Medication status regarding hypertension, diabetes, and dyslipidemia was also significantly higher in the MetS group than in the non-MetS group (*p* < 0.001 for all).

There was a significant difference in age only in the middle-aged group (*p* < 0.001) (Table 2). For middle-aged participants, the absolute leg strength of extension and flexion was significantly higher in the MetS group, but the relative leg strength of leg extension and grip strength were higher in the non-MetS group (*p* < 0.05). For the older women, the absolute strength of leg extension and flexion was higher in the MetS group, but the relative extension strength was significantly higher in the non-MetS group (*p* = 0.027). Grip strength was not significantly different between the two groups. 

Health behaviors were analyzed using the chi-square test for MetS. There was a significant difference in alcohol consumption and physical activity frequency in the middle-aged group (*p* = 0.041 and *p* = 0.011, respectively). Likewise, alcohol consumption and physical activity were significantly different (*p* = 0.046 and *p* = 0.009), but not smoking status (*p* = 0.285) for older women.

Table 3 shows the MetS ORs according to the leg strength. It can be observed that the lower the absolute strength of extension and flexion, the lower the occurrence of MetS. The prevalence of MetS decreased with leg extension (*p* = 0.005) and leg flexion (*p* = 0.003) in middle-aged patients with the lowest strength than in those with highest strength. In the older women, the prevalence of MetS decreased with leg extension (*p* = 0.004) and leg flexion in the lowest group, compared to that of the highest group. However, the lowest relative strength exhibited increased prevalence in extension of the middle-aged (*p* < 0.001) and in flexion of the older group (*p* < 0.001). The relative value of middle-aged flexion and that of the older group did not achieve a significant OR (*p* = 0.094 and *p* = 0.061, respectively).

Grip strength also showed results similar to those of leg strength. Regarding absolute grip strength, it was demonstrated that the lower the muscle strength, the lower the prevalence of MetS. Conversely, the lower the relative grip strength, the higher the prevalence of MetS. The prevalence of MetS according to relative grip strength increased in middle-aged (*p* = 0.002) and older individuals (Table 4). 

The prevalence of MetS according to health behavior was similar in middle-aged and older individuals. There were no statistically significant differences regarding smoking in either age group, and alcohol consumption and physical activity exhibited significant MetS prevalence. The prevalence of MetS in the middle-aged group increased more in the high alcohol consumption group than in the non-alcohol consumption group (*p* = 0.009) and in the older group (*p* = 0.014). No physical activity also increased in the middle-aged (*p* = 0.006) and older group compared to the 5–7 days per week group (*p* = 0.022) (Table 5).

## 4. Discussion

Cardiovascular diseases, such as ischemic cardiovascular disease and stroke, exhibit a high mortality rate worldwide; the risk increases with a high incidence of obesity, hypertension, and diabetes mellitus, and many of these risk factors are also associated with MetS [1]. Several studies have reported that the risk of cardiovascular disease is similar between men and women. A 2014 study from the UK, for example, reported that the overall incidence of cardiovascular diseases was 29% in men and 28% in women [22]. In addition, in a study on the occurrence of MetS, which analyzed data in individuals 20–39, 40–59, and >60 years old, the prevalence of MetS was higher in women than in men across all age groups [6]. A meta-analysis of 35 studies conducted in mainland China also revealed that there was no difference in the prevalence of MetS between women and men (*p* = 0.283) [23]. Rather, it was reported that the prevalence in women was higher than in men. In a relatively large Chinese study of 97,098 adults, the prevalence of MetS was 31.0% in men and 36.8% in women [24]. One of the reasons for the high incidence in women is that women tend to be more overweight and sedentary than men, with less frequent participation in physical activity [25]. Therefore, this study analyzed the prevalence of MetS in women using the strength determined by physical activity.

The results of this study regarding hand grip strength were consistent with those of previous studies. The prevalence of MetS in the women >65 years old with a high grip strength was reported as 0.54 [26], whereas men aged 35–81 years with the lowest grip strength had a 2.15-fold increase in MetS [27]. Grip strength has high reliability and validity, and effectively represents upper limb strength. It is a simple, economical measurement with portable equipment [28]. Given the many advantages of grip strength, relatively few studies have been conducted on leg strength.

The results of this study revealed a high incidence of MetS in the group with low leg strength, which is consistent with the results of previous studies. When leg strength was measured by leg press, the prevalence of MetS increased by 1.23 and 1.32 times in men under or >50 years of age, respectively, with low strength [11]. The leg strength measurement method used in most studies was either leg press or leg extension. Although rare, one longitudinal study reported a high strength of MetS. In a study that measured the one-repetition maximum of bench press and leg extension, the highest strength group exhibited a 19% lower risk of MetS than the lowest strength group [29].

While studies measuring leg strength using an isokinetic strength meter similar to the present study are rare, one such study reported that the prevalence of MetS increased 3.3-fold in older patients with low leg extension strength [10]. In addition to the prevalence of MetS, a comparative study examining the relationship between MetS factors and leg strength indicated that people with strong leg strength displayed healthy MetS indicators [30,31]. However, in previous studies, the results indicated that the leg extensors were related to MetS in men, whereas the results were not significant in women [32]. One of the features of the present study was that both leg extension and flexion strength were analyzed. While the results were significant for leg extension and flexion strength in middle-aged women, there was no significant difference in MetS according to flexion strength in the older women.

As strength is proportional to weight gain, the higher the strength, the higher the prevalence of MetS. Therefore, the relative value to which the weight is applied has been used more rationally in several studies [10,26,33]. One study conducted on 400 men further subdivided obesity factors and strength by evaluating body fat and leg extension. Compared to patients with low fat-high strength, MetS increased 8.6 times in those with high fat-high strength and 7.6 times in those with high fat-low strength. There was no significant correlation in individuals with low strength, even in those with low fat [34].

Globally, economic growth and industrial development have brought about societal changes due to increased calorie consumption and decreased daily physical activity [35]. These changes are manifested by an increase in the obese population and risk factors for cardiovascular diseases in both women and men [35]. In a Korean study comparing the periods of 2002–2003 and 2012–2013, the percentages of those overweight changed from 31.6% to 34.3% for men and 23.4% to 23.2% for women. Between these two periods, obesity increased from 2.6% to 4.2% in men and 2.9% to 3.7% in women [36]. Another study of long-term follow-up reported that the incidence of diabetes was higher in women than in men [25]. In previous studies, the incidence of obesity, hypertension, and low-density lipoprotein cholesterolemia was higher in men; nevertheless, 50% of cardiovascular mortality occurred in women [37,38]. 

This study had several strengths. First, the absolute and relative strength of grip and leg strengths were measured, and the prevalence of MetS in women was analyzed. Second, leg strength was examined using isokinetic equipment. The measurement of muscle strength using isokinetic equipment has high accuracy; it is not widely used because the equipment is expensive and the measurement procedure is complicated [39]. Therefore, the results of this study provide practical information for clinicians. To prevent MetS, it is necessary to improve relative strength. This will aid in improving the extensors of the legs as well as the grip strength, and the representative exercises are squats or leg extensions with machines.

However, our study had some limitations. Causality could not be determined because the number of participants was relatively small, and cross-sectional studies were conducted. Longitudinal studies are required to explain the causal relationship between MetS and low strength. This is because low physical activity causes MetS, but conversely, people with MetS, chronic disease or obesity can have low physical activity due to low physical strength and muscle strength [40,41]. This study did not include diet, or lean body mass. In the future, prospective studies or case-control studies are necessary to prove the effectiveness of isolated strength training for preventing or improving MetS. 

Although an in-depth analysis was not performed in this study, the impact of medication cannot be overlooked. One study reported no significant difference in the dose and exercise capacity of representative drugs such as metoprolol, hydrochlorothiazide amiloride, captopril, compared to placebo [42]. However, researchers have previously highlighted side effects of drugs on muscles and exercise. For example, statins are used to decrease cholesterol. However, one of the side effects is muscle pain, which is also called statin-associated muscle symptoms such as myalgia, creatine kinase elevation, and rhabdomyolysis [43]. It is known to occur in 10–25% of cases [44]. Typical side effects of beta-blockers used for hypertension or cardiovascular disease are fatigue, dizziness, and nausea are also widely reported [45]. Therefore, the possibility that long-term use of cardiovascular medicine could negatively impact exercise and muscle strength cannot be excluded.

Our study was conducted with an isokinetic instrument. However, the equipment is too expensive to be popularized for these tests. Therefore, the hand-held strength meter is isometric, and the method of measuring strength is an appropriate alternative. Although this method has the disadvantage of measuring at a specific angle of the joint, it has high validity compared to the isokinetic test device, is safe, is relatively inexpensive, and the measurement method is relatively simple [46,47]. In future research, it will be meaningful to conduct a large-scale study by recruiting more participants in this way.

## 5. Conclusions

The lower the absolute strength, the lower the prevalence of MetS, and the lower the relative grip and leg strengths, the higher the prevalence of MetS. In particular, the prevalence of MetS increased in women with low relative extensor muscle strength. In addition, high alcohol consumption and inactivity increase the prevalence of MetS. 

## Figures and Tables

**Table 1:** Characteristics of participants.

Variables	Middle-Aged (*n* = 453)			Elderly (*n* = 601)		
	Non-MetS	MetS	*p*	Non-MetS	MetS	*p*
*n* (%)	357 (78.8%)	96 (21.2%)	-	364 (60.6%)	237 (39.4%)	-
Age, years	48.2 ± 4.0	52.1 ± 4.8	<0.001 *	67.2 ± 4.6	67.1 ± 3.9	0.607
Height, cm	158.1 ± 4.8	158.9 ± 4.2	0.124	152.8 ± 5.3	152.9 ± 4.3	0.054
Weight, kg	56.0 ± 6.5	66.3 ± 8.8	<0.001 *	56.7 ± 7.5	61.0 ± 8.3	<0.001 *
BMI, kg/m^2^	22.4 ± 2.7	26.2 ± 3.0	<0.001 *	24.2 ± 2.5	26.6 ± 3.1	<0.001 *
MetS risk factors						
Waist circumference, cm	80.9 ± 6.9	87.3 ±7.6	<0.001 *	79.1 ± 7.5	86.6 ± 6.8	<0.001 *
SBP, mmHg	118.3 ± 14.6	132.6 ± 20.2	<0.001 *	125.9 ± 17.3	138.9 ± 16.7	<0.001 *
DBP, mmHg	78.1 ± 9.5	87.3 ± 10.5	<0.001 *	80.2 ± 9.2	89.4 ± 8.1	<0.001 *
HDLC, mg/dL	62.7 ± 13.5	42.5 ± 8.4	<0.001 *	61.1 ± 12.1	46.8 ± 10.7	<0.001 *
TG, mg/dL	95.6 ± 39.6	172.3 ± 69.0	<0.001 *	102.1 ± 43.5	168.4 ± 75.0	<0.001 *
Glucose, mg/dL	92.5 ± 8.2	104.0 ± 16.2	<0.001 *	97.1 ± 12.4	116.9 ± 12.8	<0.001 *
Medication status, (%)						
Hypertension	35 (9.8%)	24 (25%)	<0.001 *	127 (34.9%)	130 (54.9%)	<0.001 *
Diabetes	7 (2.0%)	9 (9.4%)	<0.001 *	50 (14.0%)	55 (23.2%)	<0.001 *
Dyslipidemia	25 (7.0%)	17 (17.7%)	<0.001 *	57 (15.7%)	53 (22.4%)	<0.001 *

* *p* < 0.05 by Wilcoxon Mann–Whitney test or Chi-square test; the values are expressed as mean ± standard deviation or percent (%); Abbreviations: MetS, metabolic syndrome; BMI, body mass index; SBP, systolic blood pressure; DBP, diastolic blood pressure; HDLC, high-density lipoprotein cholesterol; TG, triglyceride.

**Table 2:** Alcohol consumption, smoking status, physical activity, and absolute and relative strength of participants.

Variables	Middle-Aged (*n* = 453)			Elderly (*n* = 601)		
	Non-MetS	MetS	*p*	Non-MetS	MetS	*p*
Absolute strength values						
Leg extension, Nm	81.3± 19.1	90.8 ± 29.9	<0.001 *	62.8 ± 16.9	65.7 ± 17.7	0.029 *
Leg flexion, Nm	44.5 ± 11.3	51.8 ± 17.8	<0.001 *	31.7 ± 11.6	34.3 ± 10.7	0.003 *
Grip strength, kg	22.9 ± 4.1	24.5 ± 4.5	<0.001 *	21.0 ± 3.9	22.9 ± 3.6	0.403
Relative strength values						
Leg extension, Nm/BW	1.46 ± 0.32	1.36 ± 0.33	0.018 *	1.11 ± 0.28	1.07 ± 0.32	0.027 *
Leg flexion, Nm/BW	0.79 ± 0.19	0.77 ± 0.20	0.229	0.56 ± 0.18	0.55 ± 0.19	0.328
Grip strength, kg/BW	0.39 ± 0.09	0.37 ± 0.08	0.007 *	0.37 ± 0.07	0.36 ± 0.09	0.056
Alcohol consumption, %						
None	14.5	16.6	0.041 *	31.9	30.0	0.046 *
1 time/month	49.4	41.7		52.8	47.1	
1 time/week	26.7	27.1		10.2	12.1	
≥2 time/week	9.4	14.6		5.1	10.8	
Smoking status, %						
Never	82.2	75.1	0.381	90.7	85.0	0.285
Quit	10.2	12.5		6.1	7.9	
Present	7.6	12.4		3.2	7.1	
Physical activity, %						
5–7 days/week	15.0	12.3	0.011 *	13.9	10.7	0.009 *
3–4 days/week	47.8	29.2		37.5	29.3	
1–2 days/week	28.9	34.6		30.1	39.3	
None	8.3	23.9		18.5	20.7	

* *p* < 0.05 by Chi-square test; Abbreviations: MetS, metabolic syndrome; Nm, newton meter; BW, body weight (kg).

**Table 3:** Odds ratio of MetS according to leg strength.

Variables		Model 1		Model 2	
Middle-Aged	Group	OR (95% CI)	*p*	OR (95% CI)	*p*
Absolute Leg extension, Nm	Q1	Reference	-	Reference	-
	Q2	0.896 (0.443–1.216)	0.150	0.621 (0.037–1.323)	0.113
	Q3	0.672 (0.541–1.025)	0.125	0.545 (0.214–0.839)	0.009 *
	Q4	0.508 (0.348–0.944)	0.013 *	0.520 (0.305–0.862)	0.005 *
Relative Leg extension, Nm/BW	Q1	Reference	-	Reference	-
	Q2	1.223 (0.567–2.638)	0.413	0.666 (0.275–1.613)	0.361
	Q3	1.551 (0.570–2.324)	0.312	1.779 (1.231–3.810)	0.021 *
	Q4	2.030 (1.278–4.215)	0.009 *	2.508 (1.255–5.011)	<0.001 *
Absolute Flexion, Nm	Q1	Reference	-	Reference	-
	Q2	0.920 (0.507–1.450)	0.543	0.774 (0.030–1.184)	0.501
	Q3	0.803 (0.403–1.597)	0.219	0.602 (0.091–1.449)	0.416
	Q4	0.765 (0.386–0.918)	0.011 *	0.566 (0.130–0.885)	0.003 *
Relative Flexion, Nm/BW	Q1	Reference	-	Reference	-
	Q2	1.048 (0.800–1.408)	0.846	0.770 (0.311–2.441)	0.846
	Q3	1.187 (0.669–1.633)	0.840	1.052 (0.335–2.689)	0.540
	Q4	1.227 (0.776–2.006)	0.149	1.181 (0.703–2.279)	0.094
Elderly					
Absolute Leg extension, Nm	Q1	Reference	-	Reference	-
	Q2	0.804 (0.505–1.281)	0.459	0.794 (0.455–1.386)	0.416
	Q3	0.710 (0.463–1.088)	0.342	0.589 (0.236–1.061)	0.318
	Q4	0.639 (0.216–0.932)	0.023 *	0.583 (0.172–0.868)	0.004 *
Relative Leg extension, Nm/BW	Q1	Reference	-	Reference	-
	Q2	1.039 (0.226–1.968)	0.412	1.082 (0.512–1.314)	0.151
	Q3	1.027 (0.540–1.265)	0.241	1.435 (0.865–2.115)	0.125
	Q4	1.400 (1.099–2.181)	0.010 *	1.550 (1.096–2.148)	<0.001 *
Absolute Flexion, Nm	Q1	Reference	-	Reference	-
	Q2	0.778 (0.510–1.186)	0.741	0.880 (0.289–1.296)	0.846
	Q3	0.722 (0.273–1.852)	0.649	0.668 (0.288–1.583)	0.645
	Q4	0.619 (0.263–0.967)	0.019 *	0.562 (0.155–0.842)	0.008 *
Relative Flexion, Nm/BW	Q1	Reference	-	Reference	-
	Q2	0.669 (0.432–1.035)	0.521	0.710 (0.437–1.153)	0.479
	Q3	1.043 (0.678–1.604)	0.110	1.140 (0.445–1.830)	0.134
	Q4	1.264 (0.566–1.640)	0.098	1.283 (0.828–1.986)	0.061

* *p* < 0.05 by logistic regression; Abbreviations: MetS, metabolic syndrome; OR, odds ratio; CI, confidence interval; Nm, newton meter; BW, body weight (kg); Model 1, adjusted age; Model 2 adjusted age, alcohol consumption, smoking status, physical activity.

**Table 4:** Odds ratio of MetS according to grip strength.

Variables		Model 1		Model 2	
Middle-Aged	Group	OR (95% CI)	*p*	OR (95% CI)	*p*
Absolute Grip strength, kg	Q1	Reference	-	Reference	-
	Q2	0.723 (0.343–1.523)	0.431	0.618 (0.289–1.883)	0.411
	Q3	0.654 (0.214–1.156)	0.400	0.573 (0.314–1.149)	0.199
	Q4	0.542 (0.112–0.856)	0.019 *	0.413 (0.168–0.915)	<0.001 *
Relative Grip strength, kg/BW	Q1	Reference	-	Reference	-
	Q2	1.171 (0.564–2.431)	0.326	0.974 (0.443–2.142)	0.216
	Q3	1.251 (0.900–3.808)	0.219	1.349 (1.027–3.893)	0.015 *
	Q4	1.785 (1.107–3.549)	0.022 *	1.554 (1.178–4.314)	0.002 *
Elderly					
Absolute Grip strength, kg	Q1	Reference	-	Reference	-
	Q2	1.291 (0.840–1.982)	0.744	0.746 (0.462–1.204)	0.461
	Q3	0.668 (0.414–1.078)	0.646	0.791 (0.437–1.430)	0.240
	Q4	0.614 (0.496–1.203)	0.341	0.275 (0.162–1.464)	0.156
Relative Grip strength, kg/BW	Q1	Reference	-	Reference	-
	Q2	0.940 (0.568–1.556)	0.226	0.854 (0.504–1.449)	0.254
	Q3	1.286 (0.800–3.414)	0.164	1.108 (0.713–1.720)	0.101
	Q4	1.530 (0.926–2.208)	0.007 *	1.239 (1.052–2.124)	<0.001 *

* *p* < 0.05 by logistic regression; Abbreviations: MetS, metabolic syndrome; OR, odds ratio; CI, confidence interval; BW, body weight (kg); Model 1, adjusted age; Model 2 adjusted age, alcohol consumption, smoking status, physical activity.

**Table 5:** Odds ratio of MetS according to health behavior.

Variables		Model 1		Model 2	
Middle-Aged	Group	OR (95% CI)	*p*	OR (95% CI)	*p*
Alcohol consumption	None	Reference	-	Reference	-
	1 time/month	1.073 (0.564–2.189)	0.512	1.073 (0.431–1.985)	0.416
	1 time/week	1.399 (0.673–2.843)	0.148	1.287 (0.519–2.484)	0.110
	≥2 time/week	1.654 (1.041–3.015)	0.008 *	1.557 (1.114–3.091)	0.009 *
Smoking status	Never	Reference	-	Reference	-
	Quit	0.920 (0.611–2.145)	0.684	1.100 (0.515–1.984)	0.466
	Present	1.277 (0.719–2.641)	0.450	1.195 (0.610–2.110)	0.349
Physical activity	5–7 days/week	Reference	-	Reference	-
	3–4 days/week	1.234 (0.684–2.417)	0.149	1.034 (0.484–1.941)	0.101
	1–2 days/week	1.258 (0.584–2.613)	0.097	1.046 (0.511–2.015)	0.060
	None	2.712 (1.121–3.689)	0.021 *	2.115 (1.219–4.214)	0.006 *
Elderly					
Alcohol consumption	None	Reference	-	Reference	-
	1 time/month	1.279 (0.545–2.219)	0.845	1.079 (0.585–2.945)	0.610
	1 time/week	1.248 (0.599–2.495)	0.189	1.036 (0.594–3.155)	0.094
	≥2 times/week	2.047 (1.013–4.194)	0.040 *	1.850 (1.099–3.458)	0.014 *
Smoking status	Never	Reference	-	Reference	-
	Quit	1.343 (0.711–2.921)	0.741	1.131 (0.645–2.964)	0.501
	Present	1.835 (0.419–4.094)	0.364	1.538 (0.849–5.100)	0.284
Physical activity	5–7 days/week	Reference	-	Reference	-
	3–4 days/week	1.282 (0.511–2.671)	0.418	1.082 (0.544–2.740)	0.463
	1–2 days/week	1.352 (0.694–2.499)	0.340	1.140 (0.697–2.646)	0.290
	None	2.194 (1.085–3.974)	0.030 *	1.797 (1.102–5.109)	0.022 *

* *p* < 0.05 by logistic regression; Abbreviations: MetS, metabolic syndrome; OR, odds ratio; CI, confidence interval; Model 1, adjusted age; Model 2 adjusted age, relative grip and leg strength.

## Data Availability

The data are not publicly available because of privacy or ethics.

## References

[1] Saklayen M.G. (2018). The global epidemic of the metabolic syndrome. Curr. Hypertens Rep..

[2] Sun F., Gao B., Wang L., Xing Y., Ming J., Zhou J., Fu J., Li X., Xu S., Liu G. (2018). Agreement between the JCDCG, revised NCEP-ATPIII, and IDF definitions of metabolic syndrome in a northwestern Chinese population. Diabetes Ther..

[3] Ebrahimi H., Emamian M.H., Khosravi A., Hashemi H., Fotouhi A. (2019). Comparison of the accuracy of three diagnostic criteria and estimating the prevalence of metabolic syndrome: A latent class analysis. J. Res. Med. Sci..

[4] Thomsen M., Nordestgaard B.G. (2014). Myocardial infarction and ischemic heart disease in overweight and obesity with and without metabolic syndrome. JAMA Intern. Med..

[5] Li X., Li X., Lin H., Fu X., Lin W., Li M., Zeng X., Gao Q. (2017). Metabolic syndrome and stroke: A meta-analysis of prospective cohort studies. J. Clin. Neurosci..

[6] Aguilar M., Bhuket T., Torres S., Liu B., Wong R.J. (2015). Prevalence of the metabolic syndrome in the United States, 2003–2012. JAMA.

[7] Tran B.T., Jeong B.Y., Oh J.-K. (2017). The prevalence trend of metabolic syndrome and its components and risk factors in Korean adults: Results from the Korean National Health and Nutrition Examination Survey 2008–2013. BMC Public Health.

[8] Li Y., Zhao L., Yu D., Wang Z., Ding G. (2018). Metabolic syndrome prevalence and its risk factors among adults in China: A nationally representative cross-sectional study. PLoS ONE.

[9] Lee J.Y., Lee K., Choi Y.C. (2019). Relative grip strength cut-point and metabolic syndrome in the elderly: Korea National Health and Nutrition Examination Survey 2014–2017. J. Mens. Health.

[10] Kim H., Kim Y.H., Kim W. (2020). Association of Low Muscle Mass and Isokinetic Strength with Metabolic Syndrome. J. Mens. Health.

[11] Sénéchal M., McGavock J.M., Church T.S., Lee D.-c., Earnest C.P., Sui X., Blair S.N. (2014). Cut-points of muscle strength associated with metabolic syndrome in men. Med. Sci. Sports Exerc..

[12] Churilla J.R., Summerlin M., Richardson M.R., Boltz A.J. (2020). Mean combined relative grip strength and metabolic syndrome: 2011–2014 national health and nutrition examination survey. J. Strength Cond. Res..

[13] Arai T., Obuchi S., Shiba Y. (2017). A novel clinical evaluation method using maximum angular velocity during knee extension to assess lower extremity muscle function of older adults. Arch. Gerontol. Geriatr..

[14] Kambič T., Lainščak M., Hadžić V. (2020). Reproducibility of isokinetic knee testing using the novel isokinetic SMM iMoment dynamometer. PLoS ONE.

[15] Stark T., Walker B., Phillips J.K., Fejer R., Beck R. (2011). Hand-held dynamometry correlation with the gold standard isokinetic dynamometry: A systematic review. PM&R..

[16] Inoue S., Zimmet P., Caterson I., Chunming C., Ikeda Y., Khalid A., Kim Y. (2000). The Asia-Pacific Perspective: Redefining Obesity and Its Treatment.

[17] Mehmet H., Yang A.W., Robinson S.R. (2020). Measurement of hand grip strength in the elderly: A scoping review with recommendations. J. Bodyw. Mov. Ther..

[18] CSMi (2019). Humac Norm Users Guide.

[19] Oliveira P.F.A., Gadelha A.B., Gauche R., Paiva F.M.L., Bottaro M., Vianna L.C., Lima R.M. (2015). Resistance training improves isokinetic strength and metabolic syndrome-related phenotypes in postmenopausal women. Clin. Interv. Aging.

[20] Parcell A.C., Sawyer R.D., Tricoli V.A., Chinevere T.D. (2002). Minimum rest period for strength recovery during a common isokinetic testing protocol. Med. Sci. Sports Exerc..

[21] Bottaro M., Russo A.F., De Oliveira R.J. (2005). The effects of rest interval on quadriceps torque during an isokinetic testing protocol in elderly. J. Sports Sci. Med..

[22] Bhatnagar P., Wickramasinghe K., Williams J., Rayner M., Townsend N. (2015). The epidemiology of cardiovascular disease in the UK 2014. Heart.

[23] Li R., Li W., Lun Z., Zhang H., Sun Z., Kanu J.S., Qiu S., Cheng Y., Liu Y. (2016). Prevalence of metabolic syndrome in Mainland China: A meta-analysis of published studies. BMC Public Health.

[24] Lu J., Wang L., Li M., Xu Y., Jiang Y., Wang W., Li J., Mi S., Zhang M., Li Y. (2017). Metabolic syndrome among adults in China: The 2010 China noncommunicable disease surveillance. J. Clin. Endocrinol. Metab..

[25] Ko D.H., Lee K.H., Kim Y.H. (2020). Longitudinal study on the relative risk of type 2 diabetes mellitus according to obesity and physical activity. J. Mens. Health.

[26] Merchant R.A., Chan Y.H., Lim J.Y., Morley J.E. (2020). Prevalence of Metabolic Syndrome and Association with Grip Strength in Older Adults: Findings from the HOPE Study. Diabetes Metab. Syndr. Obes..

[27] Atlantis E., Martin S.A., Haren M.T., Taylor A.W., Wittert G.A. (2009). Inverse associations between muscle mass, strength, and the metabolic syndrome. Metabolism.

[28] Richards L., Palmiter-Thomas P. (1996). Grip strength measurement: A critical review of tools, methods, and clinical utility. Crit. Rev. Phys. Rehabil. Med..

[29] Jurca R., Lamonte M.J., Church T.S., Earnest C.P., Fitzgerald S.J., Barlow C.E., Jordan A.N., Kampert J.B., Blair S.N. (2004). Associations of muscle strength and fitness with metabolic syndrome in men. Med. Sci. Sports Exerc..

[30] Miyatake N., Wada J., Saito T., Nishikawa H., Matsumoto S., Miyachi M., Makino H., Numata T. (2007). Comparison of muscle strength between Japanese men with and without metabolic syndrome. Acta Med. Okayama.

[31] Vieira D.C.L., Tibana R.A., Tajra V., da Cunha Nascimento D., de Farias D.L., de Oliveira Silva A., Teixeira T.G., Fonseca R.M.C., de Oliveira R.J., dos Santos Mendes F.A. (2013). Decreased functional capacity and muscle strength in elderly women with metabolic syndrome. Clin. Interv. Aging.

[32] Yang E.J., Lim S., Lim J.-Y., Kim K.W., Jang H.C., Paik N.-J. (2012). Association between muscle strength and metabolic syndrome in older Korean men and women: The Korean Longitudinal Study on Health and Aging. Metabolism.

[33] Mesinovic J., McMillan L.B., Shore-Lorenti C., De Courten B., Ebeling P.R., Scott D. (2019). Metabolic syndrome and its associations with components of sarcopenia in overweight and obese older adults. J. Clin. Med..

[34] Bisschop C.N.S., Peeters P.H., Monninkhof E.M., van der Schouw Y.T., May A.M. (2013). Associations of visceral fat, physical activity and muscle strength with the metabolic syndrome. Maturitas.

[35] Egger G., Swinburn B., Islam F.A. (2012). Economic growth and obesity: An interesting relationship with world-wide implications. Econ. Hum. Biol..

[36] Min H.J., Jung W.J., Kim Y.H. (2019). Changes in obesity and physical activity according to gender in South Korean adults, 2002–2013. J. Mens. Health.

[37] Colafella K.M.M., Denton K.M. (2018). Sex-specific differences in hypertension and associated cardiovascular disease. Nat. Rev. Nephrol..

[38] Garcia M., Mulvagh S.L., Bairey Merz C.N., Buring J.E., Manson J.E. (2016). Cardiovascular disease in women: Clinical perspectives. Circ. Res..

[39] Delitto A. (1990). Isokinetic dynamometry. Muscle Nerve.

[40] Ball K., Crawford D., Owen N. (2000). Obesity as a barrier to physical activity. Aust. N. Z. J. Public Health.

[41] Kim S.E., Lee Y.S., Lee J.Y. (2020). Differences in causes of activity limitation by gender and age. J. Men’s Health.

[42] Leonetti G., Mazzola C., Pasotti C., Angioni L., Vaccarella A., Capra A., Botta G., Zanchetti A. (1991). Treatment of hypertension in the elderly: Effects on blood pressure, heart rate, and physical fitness. Am. J. Med..

[43] Deichmann R.E., Lavie C.J., Asher T., DiNicolantonio J.J., O’Keefe J.H., Thompson P.D. (2015). The interaction between statins and exercise: Mechanisms and strategies to counter the musculoskeletal side effects of this combination therapy. Ochsner J..

[44] Thompson P.D., Panza G., Zaleski A., Taylor B. (2016). Statin-associated side effects. J. Am. Coll. Cardiol..

[45] Gorre F., Vandekerckhove H. (2010). Beta-blockers: Focus on mechanism of action Which beta-blocker, when and why?. Acta Cardiol..

[46] Martins J., Da Silva J.R., Da Silva M.R.B., Bevilaqua-Grossi D. (2017). Reliability and validity of the belt-stabilized handheld dynamometer in hip-and knee-strength tests. J. Athl. Training.

[47] Romero-Franco N., Jiménez-Reyes P., Fernández-Domínguez J.C. (2021). Concurrent Validity and Reliability of a Low-Cost Dynamometer to Assess Maximal Isometric Strength in Neck Movements. J. Manip. Physiol. Therapeutics.

